# A Potential New Treatment for High-Grade Glioma: A Study Assessing Repurposed Drug Combinations against Patient-Derived High-Grade Glioma Cells

**DOI:** 10.3390/cancers14112602

**Published:** 2022-05-25

**Authors:** Sarah Lastakchi, Mary Kanyinsola Olaloko, Christopher McConville

**Affiliations:** School of Pharmacy, Institute of Clinical Sciences, College of Medical and Dental Sciences, University of Birmingham, Birmingham B15 2TT, UK; sarahlastakchi@yahoo.com (S.L.); mary.olaloko@gmail.com (M.K.O.)

**Keywords:** combination treatment, drug repurposing, primary tissue, tumour core, tumour margin

## Abstract

**Simple Summary:**

This study assessed whether a combination of drugs is more effective against recurrent grade III and IV gliomas than temozolomide, the current standard of care. This cancer is currently untreatable because, within each individual patient, the tumour consists of a genetically diverse collection of cancer cells, each responding differently to treatment using single drugs, which is a barrier to successful treatment. This study demonstrates that the local delivery of drug combinations has the potential to be more effective than temozolomide, while slowing down or eradicating tumour recurrence.

**Abstract:**

Repurposed drugs have demonstrated in vitro success against high-grade gliomas; however, their clinical success has been limited due to the in vitro model not truly representing the clinical scenario. In this study, we used two distinct patient-derived tumour fragments (tumour core (TC) and tumour margin (TM)) to generate a heterogeneous, clinically relevant in vitro model to assess if a combination of repurposed drugs (irinotecan, pitavastatin, disulfiram, copper gluconate, captopril, celecoxib, itraconazole and ticlopidine), each targeting a different growth promoting pathway, could successfully treat high-grade gliomas. To ensure the clinical relevance of our data, TC and TM samples from 11 different patients were utilized. Our data demonstrate that, at a concentration of 100µm or lower, all drug combinations achieved lower LogIC_50_ values than temozolomide, with one of the combinations almost eradicating the cancer by achieving cell viabilities below 4% in five of the TM samples 6 days after treatment. Temozolomide was unable to stop tumour growth over the 14-day assay, while combination 1 stopped tumour growth, with combinations 2, 3 and 4 slowing down tumour growth at higher doses. To validate the cytotoxicity data, we used two distinct assays, end point MTT and real-time IncuCyte life analysis, to evaluate the cytotoxicity of the combinations on the TC fragment from patient 3, with the cell viabilities comparable across both assays. The local administration of combinations of repurposed drugs that target different growth promoting pathways of high-grade gliomas have the potential to be translated into the clinic as a novel treatment strategy for high-grade gliomas.

## 1. Introduction

High grade (III and IV) gliomas (HGG) are invasive and fast-growing tumours with current treatments prolonging survival for only a few months [1,2]. The biggest challenge in developing new treatments for HGGs is the existence of tumour heterogeneity [3,4,5]. Targeted therapies have been developed and clinically evaluated in order to address molecular heterogeneity; however, all have failed to demonstrate an increase in survival [6].

Targeted therapies are disadvantaged by the presence of intra- and intertumoral heterogeneity, which consequently facilitates HGGs in developing resistance to chemotherapy [7,8,9]. The existence of heterogeneity means that there are a number of genetically different clones within HGGs, each of which is resistant to different treatments [8,10,11,12,13,14]. The pre-existence of clones resistant to treatment has been demonstrated in various types of tumours, and these clones constitute the main cause of failure of targeted therapies and are responsible for tumour relapse after treatment [15,16]. Therefore, the successful treatment of HGGs will require treatment with a combination of drugs each targeted to a different growth promoting element to ensure that all of the tumour forming clones are targeted [4,17,18,19,20,21,22,23,24].

The increase in cost, the 10 to 15 year development time for new oncology drugs, and the 12% approval rate for those drugs entering clinical development have led to the investigation of repurposing regulatory approved drugs as potential cancer treatments [17,25]. Due to these drugs having well-established dosing schedules and toxicity profiles, the cost and time of introducing them into the clinic as treatments can be significantly reduced.

In this study, four different drug combinations, made up of repurposed drugs, were investigated for their cytotoxicity and ability to reduce recurrence using a highly aggressive and hard-to-treat population of patient-derived primary HGG cells. Based on background research we selected eight drugs that would be suitable: irinotecan, pitavastatin, disulfiram, copper gluconate, captopril, celecoxib, itraconazole and ticlopidine. Due to the presence of the blood–brain barrier (BBB) and thus the need to administer high systemic doses of each of those drugs to achieve therapeutic levels at the target tumour site in the brain, we recommend that local delivery of the combinational therapy directly to the tumour margin would be the best approach. Each of the drugs were selected because they are pharmacologically well characterized; had a low likelihood of inducing local toxicity; had evidence for interfering with a recognized, well-characterized growth promoting element of HGGs [26,27,28,29,30,31,32] (Table 1); and when combined, had a reasonable likelihood of concerted activity against key biological features of HGG growth. For example, the coordinated undermining of survival paths (CUSP) protocol is a new treatment approach that combines drugs already approved for non-oncological indications as a treatment for glioblastoma (GBM), which includes captopril, celecoxib, disulfiram and itraconazole, and has shown positive results in the clinic [33,34,35]. Jiang et al. have shown that pitavastatin in combination with irinotecan is a safe and effective treatment for GBM [36]. We decided to include irinotecan in each of the combinations as we wanted to include at least one cytotoxic drug in each combination and irinotecan is the only drug, except for carmustine, that has been delivered locally to the brain [37,38,39,40]. However, carmustine has been shown to be extremely toxic for local delivery whereas irinotecan demonstrated equivalent or slightly better clinical efficacy and reduced toxicity after local administration when compared with carmustine [37,38,41]. Copper gluconate was included due to the cytotoxicity of disulfiram being dependent on copper [42], while ticlopidine was included as it is an inhibitor of the adenosine diphosphate (ADP) receptor, has been shown to inhibit ADP receptor P2Y12 in GBM and has demonstrated a synergistic effect when used in combination with imipramine [43].

Combination 1 (C1) consisted of irinotecan, pitavistatin, disulfiram and copper gluconate (Table 2). Irinotecan is a pro-drug in which active metabolite 7-ethyl-10-hydroxycamptothecin (SN-38) acts as an inhibitor of the topoisomerase I group of enzymes [44], while pitavistatin works through inducing autophagy via the LC3 pathway [36] and targeting the mevalonate synthesis pathway, which is involved in the synthesis of cholesterol [45]. HGGs depend on cholesterol for survival, and thus, its depletion would lead to cell death. Furthermore, the depletion of cholesterol in the cell membrane would enhance uptake of other drugs into the cell [46]. Disulfiram in combination with copper gluconate has been shown to be potent against HGGs with its activity dependent on the formation of the bis(N,N-diethyl dithiocarbamato)copper(II) complex [32], with a number of different mechanism of actions such as proteasome inhibition, aldehyde dehydrogenase (ALDH) inhibition, expression of serine/threonine-protein kinase (PLK1), O [6]-methylguanine-DNA methyltransferase (MGMT) inhibition or nuclear factor kappa-light-chain-enhancer of activated B cells (NFkB) activation inhibition being suggested [47,48,49,50]. This combination of drugs interferes with at least eight different growth promoting pathways of HGGs and thus has the potential to target more tumour-forming clones compared with the drugs individually.

Combination 2 (C2) is made up off irinotecan, captopril, celecoxib and itraconazole (Table 2). Captopril inhibits the matrix metalloproteinase (MMP) activity through the chelating of zinc ions at the active site of the enzyme [51]. Due to being an angiotensin-converting enzyme (ACE) inhibitor, which belongs to a class of metalloproteinases similar to MMPs, Captopril can downregulate MMP-2 and MMP-9, which are thought to play roles in GBM metastasis and invasion [52]. Celecoxib is a cyclooxygenase-2 (COX-2) inhibitor, and its anticancer effect is via the triggering of endoplasmic reticulum stress by causing leakage of calcium from the endoplasmic reticulum into the cytosol [53], the induction of p53-dependent G1 cell cycle arrest and autophagy, as well as apoptosis via NF-κB pathway [54]. A phase II trial assessing combinational treatment of oral celecoxib and intravenous irinotecan revealed some activity among recurrent brain tumour patients [55]. Itraconazole is an antifungal drug that has been shown to inhibit angiogenesis and induce autophagy through inhibition of the AKT-mTOR pathway in GBMs [56]. This combination interferes with at least five different growth-promoting pathways of HGGs, with the potential to target a number of tumour-forming clones, while also having a non-specific mechanism of action via leakage of calcium from the endoplasmic reticulum into the cytosol.

Combination 3 (C3) contains irinotecan, captopril and disulfiram (Table 2). This combination was chosen as it has been demonstrated that simultaneously inhibiting both MMP and ALDH in GBM is effective [51]. Combination 4 (C4) includes irinotecan, pitavistatin, captopril and ticlopidine (Table 2), with ticlopidine inhibiting the ADP receptor [43], and thus, this combination targets four specific growth-promoting pathways of HGGs.

In this study, we use a highly aggressive and hard-to-treat sample population and multiple tumour fragments to demonstrate that combinational treatment using repurposed drugs is more effective when compared with temozolomide (TMZ), the current standard of care.

## 2. Materials and Methods

### 2.1. Materials

Dulbecco’s Modified Eagle (DMEM-F12) basal medium (containing sodium bicarbonate and L-glutamine), foetal bovine serum, deoxyribonuclease I (DNase), trypsin replacement enzyme 1X, thiazolyl blue tetrazolium bromide (MTT), sodium pyruvate powder, di-methyl sulfoxide (DMSO), temozolomide, disulfiram and copper gluconate were all supplied by Sigma Aldrich, Dorset, UK. Antibiotic-antimycotic solution (containing 10,000 units/mL of penicillin, 10,000 µg/mL of streptomycin and 25 µg/mL of amphotericin B), collagenase, trypan blue solution (0.4%), Hank’s Balanced Salt Solution (HBSS) and Minimum Essential Medium (MEM) were all supplied by Gibco, Waltham, MA, USA. Irinotecan, pitavastatin calcium, captopril, celecoxib, itraconazole and ticlopidine were supplied by LGM pharma, Florida, USA. Pronase powder was from Roche Diagnostics GmbH, Mannheim, Germany. Ficoll-paque density gradient cushions (1.077 +/− 0.001 g/mL) was supplied by GE-healthcare life sciences, Marlborough, MA, USA. Phosphate buffer saline (PBS) was supplied by Oxoid, Hampshire, UK.

### 2.2. Biopsy Collection and Processing

Unfixed tumour core (TC) and tumour margin (TM) tissues were collected immediately following craniotomies at Queen Elizabeth Hospital in accordance with ethical approval (application number: 11-029). The samples were immediately placed in collection fluid and transported to the laboratory. Once transported, the tissue was processed immediately. The tissue was initially immersed in HBSS, sliced into approximately 1 mm^3^ fragments and then washed with HBSS to remove excess blood clots. The fragments were then suspended in 30 mL of HBSS and digested with enzymes (0.25 mg/mL collagenase, 0.5 mg/mL pronase and 0.4 mg/mL DNase) at constant low speed stirring for 2 × 30 min cycles; the solution was kept at 37 °C in the first cycle, and at 4 °C in the second cycle. Any undigested material was then sieved using a 100 μm pore nylon mesh. In 2 × conical centrifuge tubes, 12 mL of ficoll-paque density gradient cushions (1.077 +/− 0.001 g/mL) was added and equal volumes of the digested sieved suspension sample was slowly layered on top of the cushions, ensuring that the integrity of the cushions was not compromised. Both tubes were then centrifuged at 400× *g* for 30 min. Following centrifugation, the desired tumour cells formed a thin band at the interphase and were siphoned off, whilst the unwanted blood cells sedimented as a pellet which were discarded; 15 mL of HBSS was then added to the tumour cells and the solution was centrifuged for 5 min at 1200× *g*. The supernatant was removed, and the pellet was re-suspended in 1 mL of HBSS, ready for a cell viability check. Cell viability was determined using the trypan blue exclusion method, and viability scores always fell between 80 and 99%.

### 2.3. Histological Evaluation and Biopsy Protein Expression

Histological evaluation and isocitrate dehydrogenase (IDH), alpha-thalassemia/mental retardation X-linked (ATRX), MGMT and P_53_ gene mutation screening were performed at the Queen Elizabeth Hospital, Birmingham. Biopsies from patients were collected and reviewed by the neuropathologists. Tumour grade was confirmed according to the WHO classification scheme [57,58,59].

### 2.4. Cell Culture

Culture media was prepared by supplementing DMEM-F12 basal medium (containing sodium bicarbonate and L-glutamine) with 10% foetal bovine serum, 0.05 mM MEM, 1% antibiotic-antimycotic solution and 100 μM sodium pyruvate. The cells were then seeded at 2 × 10^5^ cells/cm^2^ in culture media and incubated at 37 °C and 5% CO_2_.

### 2.5. Cytotoxicity Drug Screen

#### 2.5.1. Method Enhancement Procedures Followed

To preserve the original phenotypic population as much as possible, cytotoxicity screens were only performed on TC and TM cultured cells below passages 10 and 20, respectively. MTT and IncuCyte assays were performed using 96-well plates; however, only the inner wells were used for assay, and the perimeter wells were filled with 250 μL of sterile distilled water to minimise media evaporation and thus to avoid any false positives associated from cell death due to media evaporation over the assay test period.

#### 2.5.2. MTT Colorimetric Assay

Using 96-well plates, the cells were seeded at a 4000 cells/well density, and plates were then incubated for 24 h at 37 °C and 5% CO_2_, for cell attachment. The medium was then aspirated, and 200 μL each of 3.9 (Log 0.59 nm), 7.8 (Log 0.89 nm), 15.6 (Log 1.19 nm), 31.25 (Log 1.49 nm), 62.5 (Log 1.80 nm), 125 (Log 2.10 nM), 250 (Log 2.40 nM), 500 (Log 2.70 nM), 1000 (Log 3.00 nM), 10,000 (Log 4.00 nM) and 100,000 (Log 5 nM) nM drug combination media solutions was added. Each drug in the combination was used in 1:1 ratio. Additional wells with untreated cells were used as control and medium only wells as blank. All assay plates were then transferred to an incubator at 37 °C, 5% CO_2_ for 3, 6, 8, 10 or 14 days depending on the length of treatment. Days 6, 8, 10 and 14 were only performed on the TM samples. Following treatment, 20 μL of MTT solution (5 mg/mL in PBS) was added to each well, and the plates were incubated for 3 h. The medium was then aspirated and replaced with 100 μL of DMSO to dissolve the formazan crystals formed. After 15 min, a colorimetric reading was performed using a BMG labtech FluoSTAR Omega micro plate reader (BMG Labtech, Durham, NC, USA) at a 490 nM wavelength. The percentage viability of each drug combination was calculated, and a cytotoxicity graph plotted. LogIC_50_ values were determined using Graphpad prism software version 8.00 for windows (La Jolla, CA, USA) and a Log inhibitor vs. response analysis nonlinear regression (curve-fit) model.

#### 2.5.3. IncuCyte Imaging Assay

Cells were seeded at 5000 cells/well density on 96-well plates, which were then incubated for 24 h at 37 °C and 5% CO_2_ for cell attachment. The medium was aspirated, and 200 μL each of 3.9 (Log 0.59 nm), 7.8 (Log 0.89 nm), 15.6 (Log 1.19 nm), 31.25 (Log 1.49 nm), 62.5 (Log 1.80 nm), 125 (Log 2.10 nM), 250 (Log 2.40 nM), 500 (Log 2.70 nM), 1000 (Log 3.00 nM), 10,000 (Log 4.00 nM) and 100,000 (Log 5 nM) nM drug combination media solutions was added. Each drug in the combination was used in 1:1 ratio. Additional wells with untreated cells were used as control. Subsequently, all assay plates were transferred into an IncuCyte Zoom machine (Essen BioScience, Ann Arbor, MI, USA) to incubate at 37 °C and 5% CO_2_ for 3 days, during which each well was imaged at 3 h intervals. Following the assay, images were analysed using IncuCyte zoom software (Essen Bio-Science, Ann Arbor, MI, USA). The images were masked, and cell confluence values were attained. The percentage viability of each drug combination was calculated, and a cytotoxicity graph was plotted. LogIC_50_ values were determined using Graphpad prism software version 8.00 for windows (La Jolla, CA, USA) and a log inhibitor vs. response analysis nonlinear regression (curve-fit) model.

### 2.6. Statistical Analysis

Statistical significance for the LogIC_50_ data between temozolomide and the different combination drug treatments against the equivalent sample population were assessed via paired student’s *t*-test method. Plotted data on graphs were expressed as mean ± SEM. Significance between groups was denoted by * *p* < 0.05, ** *p* < 0.01 and *** *p* < 0.001.

## 3. Results

### 3.1. Patient Demographic, Tumour Grade and Treatment History for Samples Used in This Study

Nine TC and five TM samples were retrieved from a total of eleven recurrent HGG patients consisting of eight males and three females with a median age at last resection of 57.8 years. Based on the pathology data, eight samples were classified as grade IV GBM and three were classified as grade III (Table 3). The aberrant protein expression data for IDH_1_, ATRX and MGMT were obtained for each patient, with eight patients classified with a wildtype IDH_1_ gene and nine patients classified with wildtype ATRX gene, while six patients had an un-methylated MGMT gene promoter (Table 3). This sample group is associated with an aggressive tumour that is highly resistant to treatment, with increased tumour progression and invasiveness leading to poor prognosis and shorter survival. Performing our cytotoxicity screen using a widely recognised difficult-to-treat sample population provides a more robust therapeutic screening method for the drug combinations [60,61,62].

The previous patient’s treatment record shows that patients 1, 2, 3, 6, 7, 8, 10 and 11 have had one previous resection surgery followed by radiotherapy and adjuvant TMZ chemotherapy. Patients 1, 3, 8, 10 and 11 received six cycles of adjuvant TMZ; however, they still presented with continuous progression of the tumour. Patient 2 and 6 received three cycles of adjuvant TMZ, while patient 7 received one cycle of adjuvant TMZ before it was suspended. Patient 4 and 5 both had two previous resection surgeries, and both received radiotherapy and six cycles of adjuvant TMZ chemotherapy after their first resection. Patient 4 had radiotherapy and adjuvant TMZ chemotherapy following their second resection surgery, with the TMZ suspended after two cycles. Patient 5 had radiotherapy and lomustine chemotherapy following their second resection, with the lomustine suspended after one cycle. Patient 9 has had three previous resection surgeries, and received radiotherapy and six cycles of adjuvant TMZ chemotherapy after the first two resections. After the third resection, they received radiotherapy and two cycles of lomustine chemotherapy before it was suspended. The previous treatment data confirm that this is a difficult-to-treat patient population. For example, despite receiving six cycles of adjuvant TMZ, patients 1, 3, 4, 5, 9, 8, 10 and 11 still presented with tumour progression. Furthermore, patients 5 and 9 both received lomustine after their second and third resection surgeries, respectively, which had to be suspended due to continued tumour progression. Based on their response to previous treatments, this group of patients is representative of HGG patients and is therefore an excellent sample population to use for screening these drug combinations.

### 3.2. Tumour Core Cytotoxicity Results: 3-Day Assay

**Figure 1:**
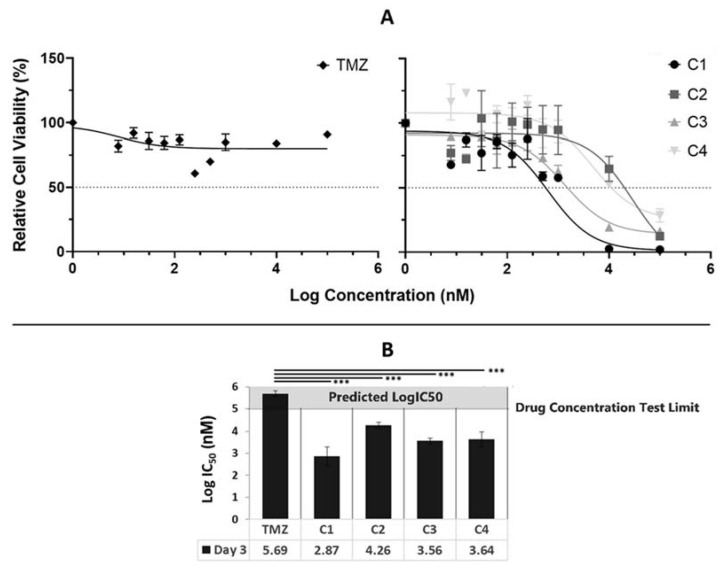
Three-day incubation average (*n* = 9) drug cytotoxicity data for TMZ and drug combinations against patient-derived tumour core samples. (**A**) Average (*n* = 9) cytotoxicity curves and (**B**) average (*n* = 9) LogIC_50_ data for nine tumour core samples treated with TMZ, C1, C2, C3 or C4. Significance between the groups is denoted by *** *p* < 0.001, if no line is drawn *p* > 0.05 within the bar charts. Abbreviations for drug treatments: TMZ, Temozolomide; C1, Irinotecan–Pitavastatin–Disulfiram–Copper Gluconate; C2, Irinotecan–Captopril–Celecoxib–Itraconazole; C3, Irinotecan–Captopril–Disulfiram; C4, Irinotecan–Pitavastatin–Captopril–Ticlopidine.

To investigate whether the C1, C2, C3 and C4 combinations of repurposed drug treatments would be more effective than TMZ, the current standard of care for HGGs, nine patient derived primary TC cells were used to perform a 3-day MTT assay. The cytotoxicity data in Figure 1A demonstrates that TMZ was unable to achieve a LogIC_50_ value in this group of patients, which is confirmed by the treatment history of this group of patients, with the majority having their TMZ treatment stopped due to a lack of response. C1 and C3 achieved cell viabilities of 8 and 25%, respectively, at a concentration of Log 4 nM while C1, C2, C3 and C4 achieved cell viabilities of 4, 12, 12 and 26%, respectively, at a concentration of Log 5 nM (Figure 1A). The average LogIC_50_ data across all nine patients (Figure 1B) demonstrate that all four combination drug treatments achieved a statistically lower LogIC_50_ value than TMZ, with C1 achieving the lowest LogIC_50_ of 2.87 nM, while C2, C3 and C4 achieved LogIC50 values of 4.27, 3.59 and 3.65, respectively (Figure 1B).

### 3.3. Tumour Margin Cytotoxicity Results after 3, 6, 8, 10 and 14 Days of Exposure

**Figure 2:**
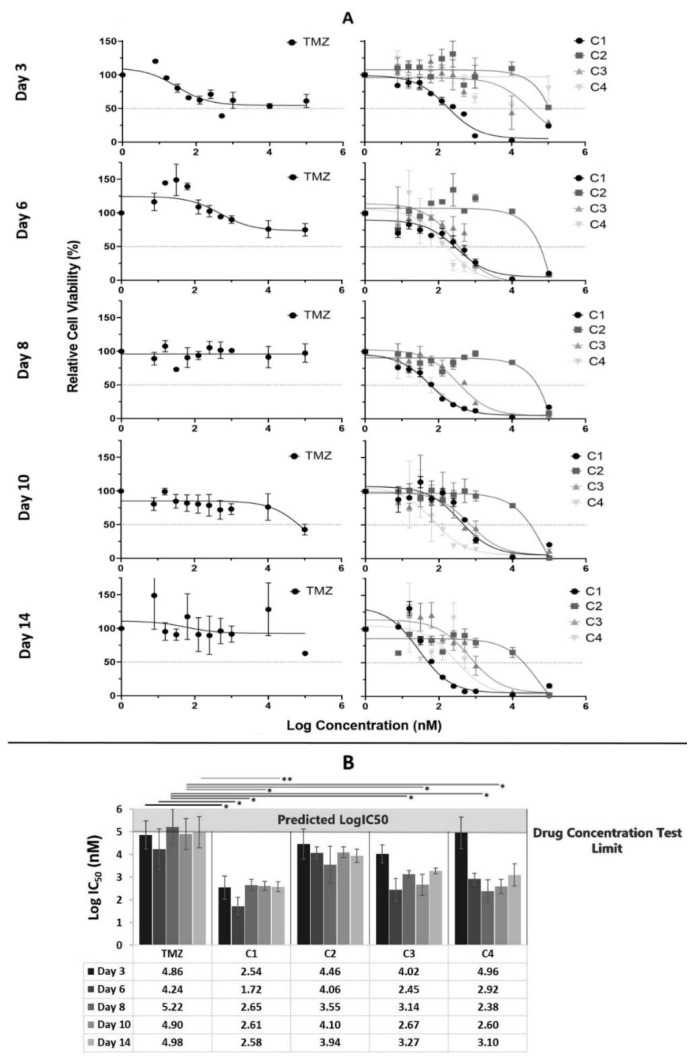
Days 3, 6, 8, 10 and 14 of incubation drug cytotoxicity data for TMZ and each drug combinations tested against patient-derived tumour margin samples using MTT assay. (**A**) Average (*n* = 5) cytotoxicity curves and (**B**) average (*n* = 5) LogIC_50_ data for the tumour margin samples treated with TMZ, C1, C2, C3 and C4. Significance between groups is denoted by * *p* < 0.05 and ** *p* < 0.01 if no line is drawn, *p* > 0.05 within the bar charts. Abbreviations for drug treatments: TMZ, Temozolomide; C1, Irinotecan–Pitavastatin–Disulfiram–Copper Gluconate; C2, Irinotecan–Captopril–Celecoxib–Itraconazole; C3, Irinotecan–Captopril–Disulfiram; C4, Irinotecan–Pitavastatin–Captopril–Ticlopidine.

Following a craniotomy, the TC is often completely removed but the TM is left behind. This residual TM is subsequently targeted via chemotherapy and radiation and can eventually develop resistance to treatment. To investigate whether combinations C1, C2, C3 and C4 would be more effective than TMZ for patients undergoing chemotherapeutic treatment after tumour resection surgery, five patient derived primary TM cell samples were used to perform MTT assays. Furthermore, to determine the impact of exposure time on the effectiveness of treatment cytotoxicity assessed at days 3, 6, 8, 10 and 14. Cytotoxicity graphs were plotted at each time point and are presented in Figure 2A while the average LogIC_50_ values are presented in Figure 2B. The cytotoxicity profile of combination C1 demonstrates that it is the most potent, with low and consistent average LogIC_50_ values ranging between 1.72 and 2.65 nM achieved for days 3, 6, 8, 10 and 14. When compared with TMZ with LogIC_50_ values ranging between 4.24 and 5.22 nM, C1 achieved a statistically lower LogIC_50_ value across all days (Figure 2B). C2 achieved consistent LogIC_50_ values, ranging between 3.55 and 4.46 nM across days 3, 6, 8, 10 and 14. However, when compared with TMZ, there was no statistically significant difference in cytotoxicity across all days (Figure 2B). With C3, lower average LogIC_50_ values were achieved on days 6, 8, 10 and 14 (2.45, 3.14, 2.67 and 3.27 nM, respectively) compared with day 3 (4.02 nM). When compared with TMZ, C3 achieved a lower LogIC_50_ value across all days; however, only days 8 and 10 were statistically significant (Figure 2B). Combination C4 also achieved lower LogIC_50_ values on days 6, 8, 10 and 14 (2.92, 2.38, 2.60 and 3.10 nM, respectively) when compared with day 3 (4.96 nM). When compared with TMZ, C4 achieved a lower LogIC_50_ value on days 6, 8, 10 and 14; however, only days 8 and 10 were statistically significant (Figure 2B).

### 3.4. Low to High Dosage Cell Viability Review

Dose escalation studies used to determine the most appropriate dose in relation to toxicity and efficacy are common practice within early stage clinical trials. To investigate at what dose each combination is most effective, we determined the average cell viability at very low (Log 2.4 nM), low (Log 2.7 nM), medium (Log 3 nM), high (Log 4 nM) and very high (Log 5 nM) concentrations.

**Figure 3:**
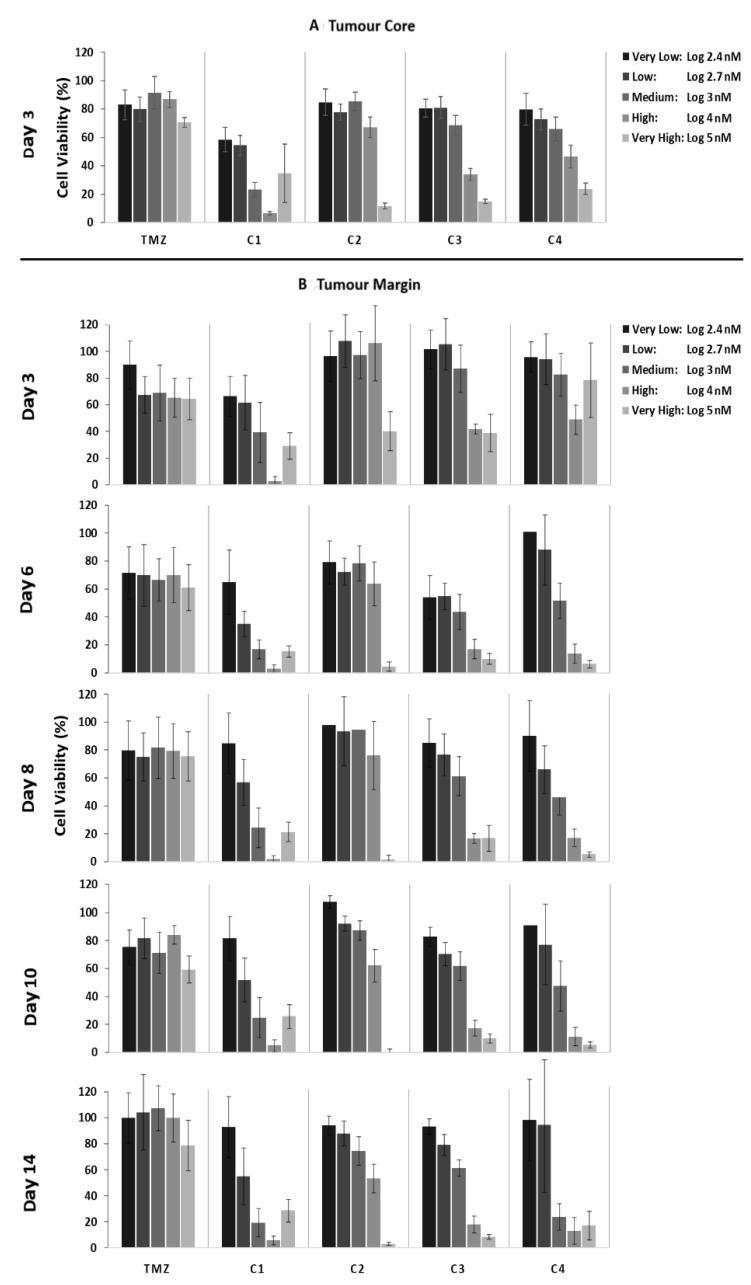
Dose evaluation study of patient-derived primary cells tested at Log 2.4, 2.7, 3, 4 and 5 nM drug concentrations using MTT assay. (**A**) Average (*n* = 9) cell viability data for the tumour core samples are 3 days of treatment with very low (Log 2.4 nM), low (Log 2.7 nM), medium (Log 3 nM), high (Log 4 nM) and very high (Log 5 nM) doses. (**B**) Average (*n* = 5) cell viability data tumour margin samples after 3, 6, 8, 10 and 14 days of treatment with very low (Log 2.4 nM), low (Log 2.7 nM), medium (Log 3 nM), high (Log 4 nM) and very high (Log 5 nM) doses. Abbreviations for drug treatments: TMZ, Temozolomide; C1, Irinotecan–Pitavastatin–Disulfiram–Copper Gluconate; C2, Irinotecan–Captopril–Celecoxib–Itraconazole; C3, Irinotecan–Captopril–Disulfiram; C4, Irinotecan–Pitavastatin–Captopril–Ticlopidine.

Data from nine patient TC fragments (Figure 3A) demonstrate that TMZ had cell viabilities between 90 and 70% across the dosage range. This again confirms the lack of efficacy of TMZ in this patient group. C1 achieved LogIC_50_ values between 58 and 6%, with the reduction in cell viabilities being dose-related (Figure 3A). A similar dose-related trend was seen for the other drug combinations, with C2, C3 and C4 having LogIC_50_ values ranging between 82 and 12%, between 81 and 15%, and between 80 and 24%, respectively (Figure 3A).

The cytotoxicity data from five patient TM fragments (Figure 3B) show that at day 3 TMZ had average cell viabilities of 90, 65, 71, 63 and 62% and very low, low, medium, high, and very high doses, respectively. There was small decrease in cell viabilities at day 6 at the very low dose and, by day 8, all cell viabilities had started to increase with all doses, except for the very high dose back to 100% viability. This is further evidence of the ineffectiveness of TMZ against this group of patients.

With C1, the average cell viabilities (Figure 3B) on day 3 were 64, 62, 40, 3 and 29% across the dosing range. At day 6, the cell viabilities continued to decrease for the low, medium, high, and very doses, while the cell viability for the very low dose remained consistent. By day 8, the cell viability for the very low dose increased to 83% and remained at this level until day 14. For the low dose, there was an increase in cell viability to 60%, which remained consistent until day 14. With the medium, high, and very high doses, there was a further decrease in cell viability to 20, 2, and 21% at day 8, were they remained consistent until day 14.

With C2, the average cell viabilities (Figure 3B) on day 3 were 95, 100, 97, 100 and 40% for the very low, low, medium, high, and very high doses. There was a further decrease in cell viabilities at day 6 across all doses. By day 8, the very low, low, and medium doses saw increases in cell viabilities, which remained consistent until day 14. However, the high and very high doses continued to see reductions in cell viabilities until day 14, with the very high dose achieving a cell viability of 2% at day 10.

C3 followed a similar trend to C2 with average cell viabilities (Figure 3B) at day 3 of 97, 99, 85, 42 and 38% for the very low, low, medium, high, and very high doses, with further decreases at day 6 across all doses, followed by increases in cell viabilities at day 8 for the very low, low, and medium doses, which remained consistent out to day 14. The high and very high doses maintained cell viabilities at approximately 16 and 12% from day 8 to day 14.

At day 3, C4 had cell viabilities of 96, 91, 80, 45 and 75% at the very low, low, medium, high, and very high doses (Figure 3B). The very low and low doses had inconsistent cell viabilities with increases at day 6, followed by decreases at day 8 and then increases at day 14. The medium, high, and very high doses resulted in decreases in cell viabilities from day 6 to day 14, with cell viabilities of 23, 17 and 19% at day 14. The very high dose achieved cell viabilities of 8% at day 8 and 10.

### 3.5. Method Validation-IncuCyte Assay

**Figure 4:**
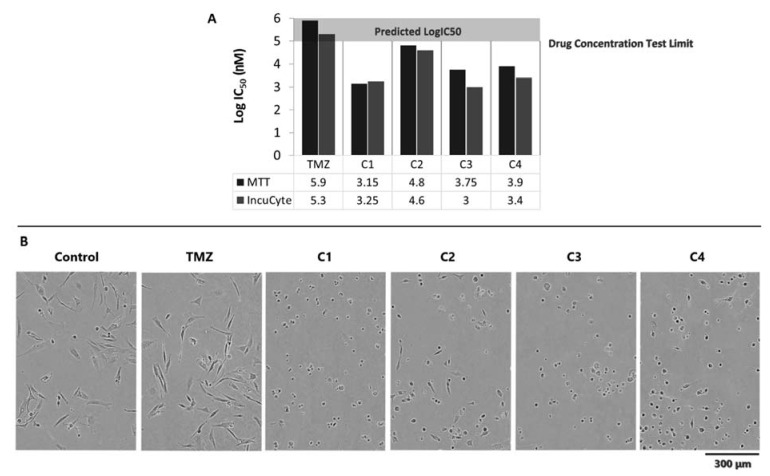
Patient-3-derived TC average (*n* = 3) drug cytotoxicity and contrast phase image data. (**A**) MTT vs. IncuCyte assay 3-day treatment cell viability data. (**B**) Control vs. Log 5 nM drug treatment phase contrast image data at day 3. Abbreviations for drug treatments: TMZ, Temozolomide; C1, Irinotecan–Pitavastatin–Disulfiram–Copper Gluconate; C2, Irinotecan–Captopril–Celecoxib–Itraconazole; C3, Irinotecan–Captopril–Disulfiram; C4, Irinotecan–Pitavastatin–Captopril–Ticlopidine.

Cell metabolism assays such as MTT rely on the cell metabolic activity as a measure of cellular viability, thus indirectly assessing cytotoxicity. Within this study, direct measurement of the cellular viability via cell imaging assay was performed on the patient 3 TC sample to validate the accuracy of the drug cytotoxicity data and as a comparison between endpoint MTT and real-time IncuCyte assays. The LogIC_50_ data for the MTT assay were 5.9, 3.15, 4.8, 3.75 and 3.9 nM, whilst the IncuCyte values were 5.3, 3.25, 4.6, 3 and 3.4 nM for TMZ, C1, C2, C3 and C4, respectively (Figure 4A). Thus, the LogIC_50_ values between the MTT and IncuCyte assay were comparable, confirming the accuracy of our data. IncuCyte control vs. Log 5 nM drug treatment cellular images are shown in Figure 4B and demonstrate that, compared with the control, the combination drug treatments C1, C2, C3 and C4 changed the cellular morphology of the cells from highly elongated to a smaller, ball-shaped structure, whilst the TMZ treated cells looked similar to the control cells, suggesting that TMZ had no impact on their morphology.

## 4. Discussion

### 4.1. Sample Test Population

The demographic of the glioma patient population in this study mainly consists of older patients with high-grade (III and IV) tumours (Table 3). This particular demographic often correlates with high resistance to treatment and lower survival rates [63].

During treatment, mutations in the IDH_1_ and ATRX genes are used to determine the grade and aggressiveness of a tumour, whereas the MGMT status is used to determine if treatment with DNA alkylating agents such as TMZ would be worthwhile. A study undertaken by Chaurasia et Al. assessed ATRX and IDH_1_ biomarker mutations and their correlation with patient survival [64]. Data from 163 patients revealed that patients with wildtype IDH and ATRX genes have worse prognosis. Hence, a combination of ATRX and IDH wildtype genes lead to significant reductions in both overall survival and progression-free survival [64,65,66]. MGMT methylation status can also impact prognosis as patients with MGMT methylation usually benefit from a favourable prognosis when treated with TMZ [67,68,69,70]. As well as MGMT methylation status, patients who have undergone a previous TMZ treatment cycle tend to be less responsive to the same treatment [71,72,73,74]. This is because recurrence is often associated with a new genetic strain that is resistant to the previous therapy.

The sample population used within this study mainly consisted of IDH_1_ and ATRX wildtype and MGMT unmethylated genes as well as patients who have previously undergone TMZ treatment (Table 3), and thus, this sample population can be classified as difficult to treat, which provides a more robust therapeutic screening method for the drug combinations.

### 4.2. Effectiveness of Combination Drug Treatment

GBM is a heterogenous cancer, and a tumour within a single patient can be described as a collection of variable genetic colonies coexisting together, with the most resistant colony existing as the dominant form within a specific time and space [8,10,75,76]. A single-drug treatment approach for a genetically diverse tumour will lead to disease progression by creating resistance to therapy over time; once the most dominant genetic form of the disease is eradicated, a less dominant form will have the chance to grow and dominate [77]. Multiple drug treatments can target different colonies to enhance the efficacy of treatment and to ultimately improve the prognosis of the patient.

Many combination drug regimens have previously been proposed to overcome the heterogenic nature of glioma tumours, with many innovative combinations being tested in preclinical and clinical trials [23,78,79,80,81]. Thus far, these have been met with limited success, often due to either high toxicity, inability of the drugs to cross the BBB or rapid systemic drug degradation [82,83,84,85]. A good example of this is disulfiram and copper gluconate combination; in a study undertaken by Senger et al., the combination was screened against patient-derived tumour cells, with the results demonstrating that the therapy impairs DNA repair pathways and enhances the efficacy of DNA alkylating agents such as TMZ [86]. Following from this study, the treatment was tested in several clinical trials using oral administration without producing any positive results [17,87]. Due to DSF’s extreme instability under physiological conditions, orally administered DSF does not reach the tumour tissue at therapeutic concentrations. Here, we assessed four carefully selected drug combinations for their use as a locally administered treatment to improve therapeutic outcome for HGG patients by targeting multiple growth promoting pathways. If rapid drug elimination and degradation can be avoided, this can reduce the dose required to obtain a therapeutic response, consequently reducing toxic side effects and as well as the development of resistance. Formulating these treatments for local administration would further minimize the dose by increasing the drug at the target site and by minimizing off-target tissue accumulation [83,88,89].

Nine TC and five TM patient-derived HGG samples were used to perform a cytotoxicity screen of the combination drug treatments using an MTT assay. All four combination drug treatments were more potent than TMZ, achieving lower average LogIC_50_ values and cell viability values across both the TC (Figure 1) and TM (Figure 2) tissue fragments.

Our data demonstrate that, at 100 µM or lower, TMZ (Figure 1 and Figure 2) was ineffective, and when we compared our TMZ data with other studies, we found that, when TMZ is tested against cell lines, the IC_50_ is between 200 and 900 µM, thus confirming our data [90,91,92,93].

On average, the C1 drug combination was the most effective, achieving the lowest LogIC_50_ values across both TC and TM patient fragments (Figure 1 and Figure 2). Based on our current understanding, this combination of drugs are known to target at least eight growth-promoting and cell-signalling pathways including topoisomerase I, LC3, mevalonate synthesis, proteasome, ALDH, PLK1, MGMT and NFkB [36,44,45,46,47,48,49,94] and has a greater effect than those targeted by C2, C3 and C4.

Following resection surgery, the TC is often completely removed but residual cancer cells from the TM are left behind and will regrow. A study performed by Andrea Sottoriva et al. revealed that, due to intra-tumour heterogeneity, separate tumour fragments can consist of different genetic mutations, and this might impact response to treatment [8]. Due to the TM fragment being difficult to obtain in sufficient quantities for tissue culture, most, if not all, patient-derived cytotoxicity assays are performed using the TC sample. However, as the TC is removed during surgery, using the TM sample to assess drug efficacy will be more clinically relevant.

To investigate whether our combination drug treatments would be effective for patients following resection surgery, five patient-derived TM samples were treated with TMZ, C1, C2, C3 and C4 for 3, 6, 8, 10 and 14 days and cytotoxicity was assessed using an MTT assay (Figure 3). While TMZ resulted in a small decrease in cell viability that was dose- and time-dependent, significant cell regrowth occurred from days 8 to 14 for all doses. This is indicative of the current treatment scenario whereby clones that are susceptible to TMZ are quickly killed off, leaving behind the more aggressive clones as well as those that are resistant to TMZ, which grow back, resulting in tumour recurrence. This tumour is then less responsive to further treatment with TMZ.

Across all timepoints, C1 achieved the lowest average LogIC_50_ values compared with all other treatments (*p* values < 0.05), thus demonstrating that it is the most effective against the HGGs (Figure 3). A key observation is that the cell viability dose was not only responsive to C1 but also time-responsive, with no tumour regrowth across the 14 days. As mentioned previously, C1 targets eight key HGG promoting-growth pathways, resulting in it targeting more HGG clones, leaving less behind to encourage cellular regrowth.

C2 and C3 had statistically similar (*p* values > 0.05) cell viability trends with both dose- and time-dependent reductions for all doses across 6 days (Figure 3). However, by day 8, the lower doses had begun to cell regrowth, which continued until day 14, while the higher doses continued to see reductions in cell viabilities until day 14. This is not surprising as C2 targets five growth-promoting pathways, while C3 targets both MMP and ALDH and, therefore, requires higher doses to reduce cell regrowth.

C4 had higher (*p* value < 0.05) LogIC50 values compared with the other combinations and an inconsistent impact on cell viabilities at lower doses, with an initial increase in cell viabilities at day 6, followed by a decrease at day 8, and then an increase until day 14, while the higher doses resulted in time-dependent decreases in cell viability (Figure 3). This is not surprising as C4 only targets four growth-promoting pathways of HGGs, and thus, the low doses result in inconsistencies as some clones are missed, while the higher doses provide enough of each drug to be effective against more clones, resulting in a consistent reduction in cell viability.

### 4.3. Dosage at Which Enhanced HGG Cell Killing (<10% Cell Viability) Is Achieved

Although reviewing IC_50_ data on its own is a very good indicator for drug efficacy, it is often worthwhile to review the individual dose vs. response data as it can provide additional information to the subtle changes in response to treatment with increased drug concentration. For example, any significant decrease (i.e., to below 10%) in cell viability above the IC_50_ concentration could also be an indicator of an effective treatment, if these concentrations can be achieved at the tumour margin.

Both C1 and C2 had significant reductions in cell viability above their LogIC_50_ value (Figure 1A and Figure 2A). C1 achieved cell viabilities below 6% across all treatment days at doses of Log 4 and 5 nM, while C2 achieved cell viabilities below 4% on days 6, 8, 10 and 14 at doses of Log 4 and 5 nM. Whilst our LogIC_50_ data revealed that C2 achieved consistent LogIC_50_ values over time, our cell viability data demonstrate that, at a dose of Log 5 nM, a reduction in cell viability over time was detected (Figure 1A and Figure 2A). This delayed response pattern was consistent across C2, C3 and C4, which all include captopril, and thus, could be due to captopril’s MMP inhibition through the time-dependent chelating of zinc ions at the active site of the enzyme [34,51]. Once this enzyme is inhibited, it allows for the accumulation of the other drugs in the combination within the cells.

Relying on IC_50_ values alone as an indicator of an effective treatment may not be suitable for treatments that will be delivered locally. With local administration, it is possible to achieve higher doses at the tumour margin and to sustain this dose for an extended period of time.

### 4.4. Data Accuracy and Validation

There are a variety of techniques to screen the cytotoxicity of therapeutic agents, with the most established and well-known being the colorimetric MTT assay [95,96,97]. The MTT assay, which is an endpoint assay, taking a single measurement after a fixed incubation period has proven to be a rapid, reliable and clinically relevant technique in screening therapeutic agents for their cytotoxicity against gliomas [96]. The IC_50_ cytotoxicity data obtained from this assay are directly dependent on the cellular metabolic conversion of MTT to formazan and thus provide an indirect measure of cellular viability. Newly emerging cellular cytotoxicity techniques rely on the real-time measurement of cellular viability. One such technique is the IncuCyte live cell analysis method, which relies on collecting snapshot images of cells as well as cell confluence data [98]. The IncuCyte analysis is a real-time assay that allows for the tracking of cellular growth over time and is particularly useful for assessing the cytotoxicity of treatments, where subtle cytotoxic effects could be missed when using endpoint-based methods [98]. Within this study, a comparison between the metabolic-based MTT and the real-time-based IncuCyte was performed on the TC sample from patient 3 to validate the accuracy of our cytoxicty data (Figure 4A). Our data demonstrate that the LogIC_50_ values across the two assays were comparable, which validates our cytotoxicity data and the conclusions made. Furthermore, using the IncuCyte assay, we obtained cellular images of our control vs. treated cells, and the images demonstrated that the TMZ cells treated with a high dose of Log 5 nM looked identical in morphology to the untreated control cells with an extended thread similar to the typical GBM structure [99,100] (Figure 4B). This is confirmation that, even at high doses, TMZ had limited impact on the primary HGG cells used in this study and confirms our cytotoxicity data and conclusion that TMZ is an ineffective treatment in this group of patients. However, the cells treated with the drug combinations appeared smaller, and less elongated and spherical, which is typical of cells undergoing cell death [99,101] (Figure 4B), which is further confirmation of our cytotoxicity data that the drug combinations elicit and the greater reduction in cell viability in this group of patients compared with TMZ.

## 5. Conclusions

In this study, we used primary HGG cells derived from both the TC and TM tumour fragments from 11 patients with known difficult-to-treat tumours to evaluate the cytotoxicity of four treatment combinations in comparison with TMZ, the current standard of care. We subsequently validated the accuracy of our cytotoxicity data, obtained using the MTT assay with the IncuCyte live cell analysis method.

Our data demonstrate that the combination therapies were significantly more cytotoxic than TMZ, achieving lower LogIC_50_ and cell viability values across all days and doses while either reducing or stopping cellular regrowth. C1 was the most effective, with the lowest IC50, greatest reduction cell viability and inhibition of cellular regrowth. This is not surprising as it targets more growth-promoting pathways than any of the other combinations. C2 was also shown to be highly cytotoxic after 6 days of exposure, with cell viabilities of less than 4%. We confirmed the accuracy of our MTT cytotoxicity data using the IncuCyte method, with similar IC_50_ values across both methods.

This data demonstrates the potential of carefully selected drug combinations as a treatment against HGGs. However, their systemic delivery could be difficult, resulting in unwanted side-effects. Therefore, we believe that the localized delivery of these combinations would allow for the administration of high local doses while sustaining these doses at the tumour margin to achieve the exposure time required for them to be an effective treatment.

## Figures and Tables

**Table 1:** The mechanisms of action of each drug against high-grade gliomas.

Drug	Mechanism of Action
Irinotecan	Inhibitor of topoisomerase I, an enzyme required for DNA transcription.
Pitavastatin	Inhibits the mevalonate synthesis pathway, which is involved in the synthesis of cholesterol, which HGGs are dependent on for survival. Furthermore, reducing the cholesterol may improve the uptake of other drugs into the HGG cells Supresses tumour cell P-glycoprotein 1, which aids the transfer of foreign substances out of the cell. Supressing this protein could enhance the potency of other drugs in the combination.
Disulfiram	Inhibitor of topoisomerase I and II, proteasome inhibition, aldehyde dehydrogenase (ALDH) inhibition, expression of serine/threonine-protein kinase (PLK1), MGMT and nuclear factor kappa-light-chain-enhancer of activated B cells (NFkB) activation inhibition.
Copper Gluconate	Growth inhibition when combined with disulfiram.
Captopril	Inhibits activity of soluble matrix metalloproteinase (MMP)-2 and MMP-9, growth-facilitating factors in HGGs. ACE inhibitors, including captopril, are well known for inhibiting angiotensin. HGGs are known to develop new blood vessels at an enhanced rate, aiding with it is migration and growth. Inhibiting angiotensin will suppress new blood vessels growth.
Celecoxib	Inhibits HGG growth by inducing DNA damage, leading to p53-dependent cell cycle arrest and autophagy.
Ticlopidine	Inhibits the purinergic receptor P2Y12. P2Y12 is expressed at higher levels in HGG cells.
Itraconazole	Induces autophagy facilitated by inhibition of AKT1 MTOR signalling pathway, which is important for regulating the cell cycle.

**Table 2:** Mechanisms of action and growth-promoting pathway targeted by each drug in the combinations.

Combination	Mechanism of Action/Growth-Promoting Pathway Targeted
C1 Irinotecan Pitavastatin Disulfiram Copper Gluconate	Irinotecan is an inhibitor of the topoisomerase I group of enzymes, while pitavistatin works through inducing autophagy via the LC3 pathway [36] and targeting the mevalonate synthesis pathway, which is involved in the synthesis of cholesterol. The depletion of cholesterol in the cell membrane would enhance uptake of other drugs into the cell. Disulfiram in combination with copper gluconate has been shown to be potent against HGGs with its activity dependent on the formation of the bis(N,N-diethyl dithiocarbamato)copper(II) complex, with a number of different mechanism of actions such as proteasome inhibition, aldehyde dehydrogenase (ALDH) inhibition, expression of serine/threonine-protein kinase (PLK1), O [6]-methylguanine-DNA methyl-transferase (MGMT) inhibition or nuclear factor kappa-light-chain-enhancer of activated B cells (NFkB) activation inhibition. This combination of drugs interferes with at least eight different growth-promoting pathways of HGGs and thus has the potential to target more tumour-forming clones compared with the drugs individually.
C2 Irinotecan captopril celecoxib itraconazole	Irinotecan is an inhibitor of the topoisomerase I group of enzymes, while captopril inhibits the matrix metalloproteinase (MMP) activity through the chelating of zinc ions at the active site of the enzyme. Due to being an angiotensin-converting enzyme (ACE) inhibitor, which belongs to a class of metalloproteinases similar to MMPs, captopril can downregulate MMP-2 and MMP-9, which are thought to play roles in HGG metastasis and invasion. Celecoxib is a cyclooxygenase-2 (COX-2) inhibitor, and its anticancer effect is via the triggering of endoplasmic reticulum stress by causing leakage of calcium from the endoplasmic reticulum into the cytosol, the induction of p53-dependent G1 cell cycle arrest and autophagy, as well as apoptosis via the NF-κB pathway. Itraconazole is an antifungal drug that has been shown to inhibit angiogenesis and induce autophagy through inhibition of the AKT-mTOR pathway in GBMs. This combination interferes with at least five different growth-promoting pathways of HGGs, with the potential to target a number of tumour-forming clones, while also having a non-specific mechanism of action via leakage of calcium from the endoplasmic reticulum into the cytosol.
C3 irinotecan captopril disulfiram	Irinotecan is an inhibitor of the topoisomerase I group of enzymes, while captopril inhibits MMP activity and disulfiram inhibits topoisomerase I and II ALDH inhibition and expression of PLK1. This combination interferes with at least five different growth-promoting pathways, while simultaneously inhibiting MMP and ALDH in HGGs has shown to be effective.
C4 Irinotecan pitavistatin, captopril ticlopidine	Irinotecan is an inhibitor of the topoisomerase I group of enzymes, while pitavistatin induces autophagy via the LC3 pathway and targets the mevalonate synthesis pathway. Captopril inhibits MMP activity with ticlopidine inhibiting the purinergic receptor P2Y12. This combination inhibits four specific growth-promoting pathways of HGGs.

**Table 3:** Tumour grade, aberrant protein expression and previous treatment for each of the patients’ tumours.

Patient	Tumour Grade	Mutation						Tumour Fragment	Previous Treatment
		IDH_1_		ATRX		MGMT			
		Wild-Type	Mutant	Wild-Type	Mutant	Methylated	Un-Methylated		
1	IV	✓		✓		✓		TM	1 RS, RT, TMZ and DEX
2	IV	✓		✓		✓		TC	1 RS, RT, TMZ and DEX
3	IV	✓		✓		✓		TC	1 RS, RT, TMZ and DEX
								TM	
4	III		✓	✓			✓	TM	2 RS, RT, TMZ, DEX, LEV
5	III		✓		✓		✓	TC	2 RS, RT, TMZ, LOM
6	IV	✓		✓			✓	TC	1 RS, RT, TMZ, DEX, LAN, LEV
7	IV	✓		✓		✓		TC	1 RS, RT, TMZ, DEX
								TM	
8	IV	✓		✓		✓		TC	1 RS, RT, TMZ, DEX
								TM	
9	IV	✓		✓			✓	TC	3 RS, RT, TMZ, DEX, LOM
10	IV	✓		✓			✓	TC	1 RS, RT, TMZ and DEX
11	III		✓		✓		✓	TC	1 RS, RT, TMZ, LEV

## Data Availability

Access to the data is available by contacting C.M. directly.

## Abbreviations

IDH_1_, isocitrate dehydrogenase 1; ATRX, alpha-thalassemia/mental retardation X-linked; MGMT, O-6-methylguanine-DNA methyltransferase; TC, tumour core; TM, tumour margin; RS, resection surgery; RT, radiation therapy; TMZ, temozolomide; DEX, dexamethasone; LEV, levetiracetam; LOM, lomustine; LAN, lansoprazole.
